# Experiences of hospital health professionals in the care of deaf patients

**DOI:** 10.15446/rsap.V25n6.109176

**Published:** 2023-11-01

**Authors:** Natalia Ruiz-Arias, Rene M. Barría-Pailaquilén

**Affiliations:** 1 NR: RN. M. Sc. Nursing Science, Universidad Austral de Chile. Valdivia, Chile natalia.ruiz.22@gmail.com Universidad Austral de Chile Universidad Austral de Chile Valdivia Chile; 2 RB: RN. M. Sc. Clinical Epidemiology. Ph. D. Public Health. Universidad Austral de Chile. Valdivia, Chile rbarria@uach.cl Universidad Austral de Chile Universidad Austral de Chile Valdivia Chile rbarria@uach.cl

**Keywords:** Deafness, patients, patient care, delivery of health care, quality of health care, qualitative research *(source: MeSH, NLM)*, Sordera, pacientes, atención al paciente, atención a la salud, calidad de la atención de salud, investigación cualitativa *(fuente: DeCS, BIREME)*

## Abstract

**Objective:**

This study aimed to describe the experience of hospital health professionals in the care of deaf patients.

**Methods:**

We explored primary qualitative data from the experience of hospital health professionals in caring for deaf patients. A qualitative study with a descriptive phenomenological approach was carried out using face-to-face semi-structured qualitative interviews (N=8). We evaluated the perspective and experience of healthcare professionals caring for a deaf patient through semi-structured interviews (supported by field notes) which were audio recorded, transcribed, and analyzed using the Colaizzi Method.

**Results:**

Ninety-four significant statements emerged and were grouped into two themes: "professional-patient interaction" and "conditioners of care", each with three categories. The interactions were influenced by factors such as the tools and strategies used by the health professional, their emotions, and the patient's conditions; on the other hand, within the conditioning factors, the professionals described the procedural aspects, environmental and structural factors linked to care and access.

**Conclusions:**

The health professionals' experience impacts both themself and the quality of patient care. This impact is associated with different barriers, such as interpersonal, interaction, cultural and language or communicational, which generates inequality in access to health for deaf people and a challenging situation for health professionals.

## RESUMEN

**Objetivo:**

Este estudio tuvo como objetivo describir la experiencia de los profesionales de salud hospitalarios en el cuidado de pacientes sordos.

**Métodos:**

Se realizó un estudio cualitativo con enfoque fenomenológico descriptivo mediante entrevistas cualitativas semiestructuradas cara a cara (N=8). Evaluamos la perspectiva y la experiencia de los profesionales de la salud que atienden a un paciente sordo, por medio de entrevistas semiestructuradas (apoyadas en notas de campo) que fueron grabadas en audio, transcritas y analizadas mediante el método Colaizzi.

**Resultados:**

Emergieron 94 enunciados significativos que fueron agrupados en dos temas: "interacción profesional-paciente" y "condicionadores del cuidado", cada uno con tres categorías. Las interacciones se vieron influidas por factores como las herramientas y las estrategias utilizadas por el profesional de la salud, sus emociones y las condiciones del paciente; por otro lado, dentro de los condicionantes, los profesionales describieron los aspectos procedimentales, ambientales y estructurales vinculados al cuidado y al acceso.

**Conclusiones:**

La experiencia de los profesionales de la salud impacta tanto en ellos mismos como en la calidad de la atención al paciente. Este impacto se asocia con diferentes barreras, como las interpersonales, de interacción, culturales y de lenguaje o comunicacionales, lo que genera desigualdad en el acceso a la salud para las personas sordas y una situación desafiante para los profesionales de la salud.

According to the World Health Organization (WHO), in 2021, over 1.5 billion people experience some degree of hearing loss, and by 2050, nearly 2.5 billion people are projected to have some degree of hearing loss 1. In Chile, according to the National Disability Survey (ENDISC), in 2004, 1.8% of the population was deaf or hard of hearing 2, while for the year 2012, this increased to 2.9% 3.

Today, disability is recognized as a social problem influenced by personal and environmental factors. For example, according to research done in the UK, 8 out of 10 deaf people want to use sign language, but only 3 out of 10 have the opportunity to do so. In addition, other methods, such as lip-reading, may be hampered by circumstances such as poor lighting, the interlocutor having a moustache or beard, mumbling, rapid speech, or yelling, etc. 4.

The WHO states that hearing loss has been referred to as an "invisible disability", mostly because it has long been stigmatized in communities and ignored by policy-makers 1. Since the "hearing world" poorly understands deafness, they often fall into erroneous assumptions, prejudices, and stereotypes. The enormous and widespread lack of knowledge about deafness is documented in society and healthcare. In the latter, health professionals cannot always approach deaf people with the correct strategy, affecting the results of care and the quality of the service delivered 5.

Deaf people feel unable to participate appropriately in their healthcare 6. According to studies, the lack of effective communication between deaf patients and health professionals can have serious consequences. Misdiagnoses, mistaken treatment, and medication failures due to incorrect or incomplete medical instructions are the greatest risks to the deaf community, in addition to unnecessary tests and difficulty in scheduling a medical appointment 6.

The greatest difficulties faced by deaf people are related to the problems of language acquisition and the development of a communication system 4, which is why deaf people have difficulties integrating into different areas of daily life, such as education and health, among others. Some deaf patients have described difficulty accessing health care and recognize their healthcare encounters as often meaningless. This leads to frustration with the healthcare system and evidence of the lack of resources available to these patients 7. Other studies report that some deaf patients have perceived health professionals as culturally insensitive, explaining that doctors repeatedly fail to maintain eye contact (or face to face contact) and enunciate when communicating with deaf people 8. Based on these issues deaf people frequently give up trying to explain themselves to health professionals and are more likely not to ask for additional information about their health 8.

In the US, the prevalence of hearing loss is greater than other chronic diseases 9, which are well covered and studied in most health professions curriculum, such as heart disease, asthma, or diabetes. For this reason, Barnett compares diabetes as an impairment that affects glucose metabolism with the concept of hearing loss as an impairment that affects communication 10. The difficulties that health professionals present to communicate, such as the lack of knowledge about the patient's culture, language, and literacy level, can influence the level of care delivered to the patient 11.

Healthcare professionals who fail to communicate to patients with language difficulties fail to act under their ethical and professional duties and face potential lawsuits for malpractice 6. Therefore, health communication literacy aids the ability of people to understand and use information from reliable sources 12.

Health care for deaf people could be improved if health professionals had fundamental and updated knowledge about the socio-cultural and language aspects of deafness. Also, conditions would improve if the barriers to health care for the deaf community are removed, services are upgraded, and adequate resources are provided 8. Unfortunately, in Chile, the problem of approaching deaf patients is not explicitly included in curricula for health professionals in universities.

Research related to the issues that the deaf community faces focus mainly on their accessibility to the health system, but very few focus on the health professional and their capacities. To date, few studies have been carried out in Chile and Latin America, with a large number of the population in these territories suffering from this disability.

Therefore, we aimed to describe the experience of hospital health professionals in the care of deaf patients, and thus, try to reduce communication barriers and prevent disability from becoming a limitation.

## METHODS

### Study design

We chose a descriptive phenomenological qualitative design, the most suitable for exploratory research. Phenomenology is a method that allows researchers to understand the fundamental structures of different experiences about a common topic 13, and it is helpful in investigating previously unknown or, in this case, overlooked experiences 14. This design considers the health professional's perspective, their experience on a particular phenomenon, the impact of contextual factors, and the complexity of interactions 15.

### Context, participants, and sampling

The study was carried out between August 2019 to January 2020 at a highly complex tertiary hospital in Valdivia, Chile. We included eight different health professionals such as doctors, nurses, physiotherapists, speech therapists, etc., both from outpatient units and hospitalization units (Table 1), who have experienced caring for deaf patients or dealing with deaf guardians/parents in the case of pediatric care.

**Table 1:** Characteristics of participants and settings

Interviewee	Profession	Gender	Department (Setting)
#1	Doctor	Male	Pediatric Emergency Room
#2	Doctor	Male	Otorhinolaryngology Polyclinic
#3	Dentist	Male	Department of Dentistry
#4	Nurse	Female	Department of Orthopedics and Traumatology
#5	Physiotherapist	Male	Department of Orthopedics and Traumatology
#6	Speech therapist	Female	Intensive Care Unit
#7	Nurse	Female	Department of Oncology
#8	Nutritionist	Female	Department of Urology

We used a purposive non-probabilistic sampling of maximum variation 16, to obtain heterogeneous view of the participants who have experienced the phenomenon under study. There was no discrimination criterion for the participants; in such a way, professionals from the health area of different units, ages, and professions were inter-viewed. No professionals refused to participate.

The number of participants incorporated into the study complied with the criterion of saturation sampling 17,18. This means that we sampled until data replication or redundancy occurred. We believe that sufficient saturation was reached, consistent with the chosen thematic analysis proposal 19.

The researchers had no prior relationship with the participants. Before carrying out the data collection, we obtained informed consent. We explained the aim of the study and aspects of participation, the reasons, and the interest of the researchers on the research topic. In this first personal interaction, the experience of the researchers in this type of study was also shared. All this facilitated the development of rapport 20.

### Data collection

Data were collected through face-to-face semi-structured interviews conducted by a trained researcher (NR). The interviews were recorded in digital format (WMA) using a recorder (Olympus Digital Voice Recorder VN-5200PC) and followed a guide that included the questions: How have cared for deaf patients? How was your relationship with the patient? What do you think are the factors that limit or facilitate care? All participants were encouraged to talk freely and use their own words to tell their stories 21. Since we used semi-structured interviews, we included the possibility to add new questions while the conversation was taking place. All the participants were interviewed at their workplace at the end of their shift as not to interfere with their work schedule. The interviews lasted between 30 and 40 minutes, and no subsequent interviews were necessary. At the end of each interview, the researcher reminded the participants about the necessity for follow-up contact by email to discuss the final study results, ensuring, that the study findings reflect each experience 21. Finally, the literal words were transcribed from the recording, while non-verbal communication (expressions) and the context were included from notes made during the interview.

### Data analysis

The Colaizzi Method was used independently by both investigators for the analysis, using the seven steps that characterize it 22. These clear, logical, and sequential steps are applicable and useful in phenomenological research to improve the reliability and dependability of the study findings 23. For this reason, this method allows the interviews to be analyzed in detail since it begins with the familiarization step, which corresponds to the researcher reading the interviews and then identifying significant statements, formulating meanings, grouping these into categories and emerging themes. Thus, the phenomenon is exhaustively described from the interviews, then condensed into a fundamental structure and presented back to the participants, who will then approve or reject the description 21. This last step makes the difference between this method and other phenomenological data analysis methods because the participants must corroborate the findings to guarantee they are accurate and credible 23.

### Rigor

To meet the methodological rigor criteria 24, we developed the following actions. To achieve credibility, we recorded the interviews, and then we made the exact transcription of the speeches. We also used field notes; Subsequently, we contrasted the interviews and findings with the interviewees themselves, who validated the results. We also performed triangulations between researchers to evaluate the consistency of the results. To meet the auditability and applicability criteria, the methodology used, the characteristics of the informants and the context have been described in detail.

### Ethical considerations

The study was approved by a Research Ethics Committee and meet the ethical requirements for human research.

All procedures performed in studies involving human participants were in accordance with the ethical standards of the Helsinki Declaration and Belmont Report. Informed consent was obtained from all participants included in the study.

## RESULTS

**Figure 1:**
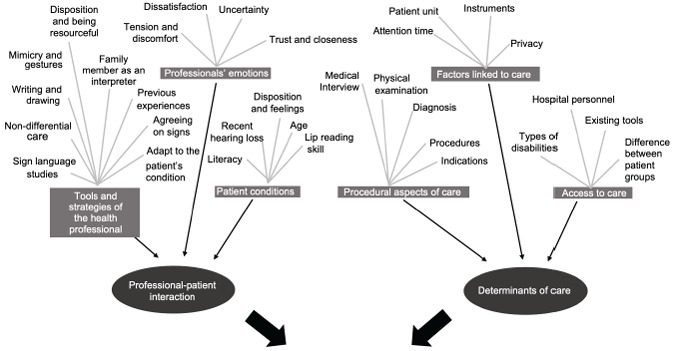
Thematic map

Eight interviews were conducted with professionals from 94 significant statements were extracted, which were grouped into two themes, each with three categories (Figure 1). Illustrative verbatim quotations were chosen to demonstrate the main categories and themes (Table 2).

**Table 2:** Illustrative quotes for the themes and categories

Theme	Category	Quotes
Professional-patient interaction	Professional tools and strategies	I try to explain what I am going to do with gestures, I show them some things so that they understand what will be done in this session. I gesture more, I try to draw a picture, I try to explain, putting more emphasis on each step. (#3). Some students taught her to express when she was in pain and when she was not, so we understood each other like that. (#7). You have to be willing to make the final change or provide the required time. (#7). Another thing that has a lot of influence is the predisposition of the staff when trying to understand them. (#6). They told me "you understand them", or they communicate well with you. There are other people who find it more difficult, they have had less experience. (#4). In undergraduate I tried to do a sign language course, to be able to communicate with them, I didn't finish it [...], but I did learn a lot, so I learned to communicate well with them or at least be able to understand what they want and to be able to express myself too (#5). The first thing I try is to try do is let them know that I can understand them and that they can express themselves and make everything they feel known. (#5). For me, that cannot be, the patient has to eat, and eat well, that is, I adapt to their condition, not that they adapt to mine. (#8). ... Over the years, these patients have acquired the skills to communicate in some other way. Although some cannot even sign language, they often manage to communicate with a family member and perfectly understand each other. In that case, the family member acts as a translator or interpreter. (#2). In the end, the family members often end up making decisions for this patient because we are unable to explain what they have. (#6).
	Professionals' emotions	The moment the person tells you that they do not speak, one already puts themselves in a quite tense situation. (#1). What a pity that I could not answer the concerns [...] and one leaves feeling that the job is unfinished. (#1). When you understand them or manage to understand them, they become much closer to you and trust you much more than other patients. A stronger bond is generated from them to us as professionals. (#5).
	Patient conditions	Between gestures and lip movements, because he read lips, he understood perfectly. (#4). I think that younger patients are easier to work with, they understand faster, so it seems. (#3). Our population is also highly rural, of high social risk. This creates a bias when we have a patient who comes from the countryside, has been with their mother their whole life, who understands them, and that is where you start running out of tools. (#6). The main problems have been in patients who have lost adult hearing, who have had language all their lives and lost hearing from one moment to the next, so these patients never acquired the ability to read lips, or to communicate with certain gestures. (#2). The feeling of not wanting to bother, which is generally greater in these patients, this situation is a little more present due to difficulties in communicating. (#5).
Determinants of care	Procedural aspects of care	Specific details such as if it hurts all the time or if the pain is oscillating, that I could not ask, the rest I could handle almost completely with the physical examination. (#1). When it comes to other pathologies that are not necessarily related with the auditory issue, it is difficult to obtain clarity, it is much slower to arrive at an adequate anamnesis, and therefore reach a diagnosis. (#2) With these patients, one has a certain degree of difficulty, and sometimes one even falls into this situation where the patients are feeling some degree of discomfort, due to the inability to communicate to explain you have to do with each case. (#2). Sometimes you do not know if the patient understood the instructions you gave, and you are left with that doubt. (#3).
	Factors linked to care	I couldn't say how it is in other situations, but at least I have the disposition to do things, but even if the rest are there circulating and they see that you are fluttering, that you are moving, perhaps one gets even more nervous. (#1). It is necessary to turn on the lights [to communicate with the patient], the good thing is that each bedside has an individual light for patients at night. We also taught them that, when they wanted to call us, they could ring the bell. (#4). We have a blackboard with a pencil, and therefore he erased and asked us about his exams, how everything was, what was going to happen with him ... (#7). We have to dedicate a little more time because obviously, the communication tools are more limited. (#5) Sometimes when a patient arrives with profound deafness, one does not have the time to be exclusively dedicated to the patient, but the patient requires more time, which generates a delay. (#2)
	Access to care	Today, for the Haitian immigrant patient, we have been taught a little Creole [...], but we have been treating deaf and hard of hearing paitents for years, and there are few, if not zero, opportunities for the staff to learn sign language, even the basics. (#6). In Creole, for example, you go to Google, to the translator, and you can do it or in part at least, here [with deaf patients] I have nothing to support myself. (#1). So each level poses its challenges, it is not only the responsibility of the direct clinical staff [...] but also of all the staff who assist them. (#6).

### Theme 1: Professional-patient interaction

#### Professional tools and strategies

The professionals reported the different methods they use to communicate with a deaf patient at the time of care, for example, signs, gestures, mimicry, writing, drawings, articulating words so that the patient can lip read. In addition, they mentioned attitudes that helped them when caring for a deaf patient, such as disposition and being resourceful. Having previous experiences and having studied Sign Language at university were factors that made it easier for the professional to understand the patient, and the importance of letting the patient know that they can express themselves freely was alluded to. It was mentioned that providing non-differential care between patients regardless of their abilities is essential and that the professional must adapt to the patient's condition, not the patient to the professional's condition. And finally, the most mentioned tool was the family member as a translator or interpreter. Since many of the patients have not acquired some kind of formal language to communicate, the family members understand them best and usually accompany them.

Due to the communication barrier that prevents the patient from explaining actions and procedures, family members must participate in decision-making regarding the patient's diagnostic and therapeutic procedures.

#### Professionals' emotions

All of the above generate different emotions in professionals, such as tension and discomfort, and seeing their tools to communicate limited, and they describe feelings of dissatisfaction and uncertainty. Additionally, once communication was established, the professional reports that deaf patients feel much more trust and a stronger closeness is generated than with other patients.

#### Patient conditions

The most mentioned characteristic that facilitates communication was the ability of the patient to read lips. Patient age was also considered an important factor since communication with younger patients is easier for the professional. On the other hand, professionals describe that there is an age range of deaf patients who are not literate (over 45-50 years), added to the fact that the patient is from a rural sector, with a lack of education, therefore, this makes care difficult for the professional because it limits the strategies that could be used, since a large part of this population cannot read or write, and they do not use Chilean Sign Language. When a deaf person, who recently lost their hearing, has not acquired any type of formal language, such as signs, lip-reading, etc., it makes it much more difficult for the professional to find communication tools during care. Also, the patient's bad disposition or feelings of insecurity and anguish also influence the care.

### Theme 2: Determinants of care

#### Procedural aspects of care

Due to the communication barrier, it is difficult for the professional to define the reason for the consultation, and, likewise, the strategies to specify the interview are insufficient. Others mention that it is not even possible to complete the interview, so they prefer to continue with the physical examination to collect more data. This is why the physical examination becomes a fundamental tool when developing an adequate anamnesis is impeded. Also, there are details of the care that cannot be specified, which makes it difficult to determine a diagnosis. Additionally, many professionals are unable to explain the procedures they are going to perform. Finally, the professionals feel that the patient is not satisfied with the care, and that, despite their efforts, the patients do not fully understand the indications.

#### Factors linked to care

The professionals described environmental and structural factors, both physical and temporal, which can influence communication with the deaf patient, such as the privacy of the place where the care is performed, which varies in the context of hospitalization, from ambulatory care and urgent care. The presence of people outside the professional-patient interaction generates discomfort when establishing communication, and the patient unit also plays an important role, according to some professionals. Having instruments to facilitate explanations, such as a blackboard, and allowing the patient to write, facilitates communication with the patient and creates a more dynamic process. Moreover, a recurring condition described in different interviews was that the attention time is insufficient due to the communication barrier generated by the lack of professional tools. 

#### Access to care

The interviewed professionals stated that higher priority is given to the communication barrier that is generated with immigrants and native Chilean people, and not to the barrier that is generated with deaf patients, even though it is a population that has always been present. Professionals do not know which tool to turn to when dealing with this type of patient, unlike patients who speak other languages, since, in the case of an immigrant patient, they report that it is enough to use cybernetic tools to communicate. There are no such tools with deaf patients. Also, they refer that it is a problem that is extrapolated to all types of disabilities, not only to deaf patients; and that within the health area, more emphasis is placed on child disability, even though there are patients with disabilities of all ages. Additionally, a communication barrier is generated with all hospital personnel, not only with health professionals.

## DISCUSSION

This study has made it possible to approximate the communication phenomenon in health care between professionals and deaf patients. We analyzed aspects related to the professional's interaction with patients and access to care and how they influence the professional's experience.

Professionals must look for tools and strategies to communicate when caring for a deaf patient, since there are no previously established strategies or protocols to attend to them. In previous studies, it was mentioned that even without knowing Sign Language, it is necessary to have skills to interpret gestures, facial, and body expressions 25. There are anti-discrimination laws that protect the deaf community in other countries, for example, "Equality act" in the UK, "Health and Medical Service Act" in Switzerland, 'Act of Equal opportunities for disabled persons" in Germany, "The American with Disability Act" in the United States, among others; the latter establishes that all health care facilities and providers in the United States must provide reasonable communication accommodations, including qualified Sign Language interpreters, when dealing with deaf patients 6. In Brazil, the Ministry of Health has developed a manual for doctors, nurses, and different health professionals, intending to include people with disabilities in the context of health. Specifically, Decree No. 5626 of Law No. 10.436/02 contains points such as training health professionals in the use of Sign Language and that deaf patients can be cared for by these professionals. Thanks to this law, public institutions are responsible for providing training to health workers for the care and treatment of these patients 26. There are also international conventions that protect people with disabilities, such as "Charter of Fundamental Rights of the European Union" and "UN Convention on the Rights of Persons with Disabilities" 6.

In Chile, Law No. 20.422 establishes rules on equal opportunities and social inclusion of people with disabilities. Within article No. 3, the concept of Universal Accessibility is defined as the condition that all products, services, instruments, tools, and devices must be understandable and usable by all people, autonomously and the most naturally possible 27. Within the health area, Law No. 20.584, established the rights and duties of the patient, where we can find the right to receive understandable information about their health status, respect for privacy, and the existence of signage and facilitators in the original language where relevant 28; but the latter, according to the law, only applies to indigenous population.

According to the 2012 census, more than 60% of the Chilean population is located in the lower two socioeconomic strata (D and E). These strata are characterized by not having complete education or university studies, low-paying jobs, and belonging to the public health system 29. Given that in Chile, deaf people have lower incomes, fewer years of education, and higher unemployment rates than the general population 30, making access to private health care less probable.

Barnett says that the key to effective communication with deaf people or people with hearing loss is adapting to the situation. Patients often have good suggestions on how to best communicate with them; therefore, it is vital to consider and incorporate their help. This ability can be trained and may help students and health professionals work more effectively with deaf patients 10.

Professionals who reported having studied Sign Language at university mentioned having greater ease in communicating with patients. This was previously proven in a study of medical students in the UK, which included workshops on Sign Language and communication skills with deaf people. This study showed that after the workshops, the students had a better attitude towards deafness. The same students suggested that it would be valuable to integrate some training to raise awareness about deaf patients, including communication skills 31.

The family member as an interpreter stands out as one of the most relevant tools, which facilitates communication with the professional. This situation was mentioned in previous studies and, although it is an element that contributes to communication, it affects other spheres within the interaction with the professional, such as patient autonomy and the right to privacy. For example, due to the lack of effective communication, deaf people attend appointments accompanied by someone to help them, usually a hearing relative who they can trust. However, the participants of this study reported that during the consultation, the companions hardly shared the information exchanged with the professional, so these people tend to make decisions about the body and health of the deaf person, discouraging their autonomy 32. There are communication barriers that frequently complicate the interaction between the deaf person and the health professional, making interpreters often required. Whether they are family members or certified professionals, this inevitably leads to a clear risk of violation of the right to privacy of health information, despite the consent of the deaf person for the interpreter to participate in the interaction 33. Additionally, the age and degree of literacy of the patient are conditions that influence communication during care. This was previously described as part of the access barrier to information for deaf patients and referred to differences in the ability to obtain information, mainly due to age, which reflects the access they had to education and the environment in which they grew up 33.

The professional's perception of the patients' feelings and attitudes during care makes communication limited by not clearly exposing the patients' questions. It was previously described that patients can lose essential parts of the conversation by not hearing the words, seeing the speaker's face, or understanding the written words, making them insecure because they do not want to appear annoying. Therefore, patients pretend to understand what the professional communicates 34.

Different problems related to the procedural aspects of care are identified due to the communication barrier, such as difficulties in the assessment, physical examination, diagnosis, procedures, and indications. These difficulties during care can cause consequences for the patient. In this way, poor communication can have repercussions on the health and safety of the patient since, if there is inadequate communication, diagnoses can take longer or be less precise. Also, inappropriate or erroneous treatments can be initiated, as the patient may misinterpret the medication dose and may ingest a highly dangerous or insufficient dose. In other cases, lack of communication means that the deaf person does not understand the medical intervention they are under-going or the final diagnosis 1,11.

Due to the conditioning factors of care, such as access, the term *invisibilization* arises. This term refers to the set of cultural mechanisms that omit a particular social group 35. The results show a certain degree of invisibility in this group (deaf people) compared to other groups such as foreigners and indigenous people. Cultural and language barriers in access to health are factors that result in poor health conditions in patients and a decrease in the use of services. Additionally, knowledge and training on cultural diversity permit understanding the patient's culture; however, these efforts focus on racial and geographic cultures (34). As mentioned by the interviewed professionals, higher priority is given to the communication barrier generated with immigrants and indigenous peoples in Chile. In the case of disability, these training sessions focus on childhood disability. Currently, there is a health care manual for deaf people, prepared by the Department of Speech Therapy of the University of Chile, freely distributed, which seeks to guide professionals in primary health care centres 36.

### Limitations

To adequately assess the findings of this study, we must take into consideration some limitations. Although we achieved data saturation with the eight interviews and met the criteria of methodological rigor, the interviewed population did not encompass the entire range of health professionals, such as midwives, medical technologists, and psychologists. Additionally, the study only covered the hospital context (both emergencies, hospitalization, and outpatient care); therefore, it cannot reflect what happens in Primary Health Care.

However, with the limitations, this study has exposed the current gap in health care for disabled people, showing the need to break down the existing barriers by improving current strategies and tools and creating alternatives for equitable access to health for the entire population.

This study presents the inequality in healthcare access. The number of professionals who had a history of treating a deaf patient is very low; therefore, it is inferred that the deaf population is not using health services. Research indicates that the professionals' experience affects both themselves and the quality of patient care. This impact is associated with the different barriers that were identified during the study, such as interpersonal, interaction, cultural and language or communicational, which generates inequality in access to health (both information and services) for deaf people and a challenging situation for professionals. The professionals who mentioned having had some training during undergraduate showed greater ease in communicating with patients and providing adequate care.

### Implications of results for practice

Deaf patient care requires that health professionals have the skills and competencies to provide timely and efficient health care and thus avoid non-attendance for controls or that these patients do not attend to treat their needs. Health centers must facilitate the access and stay of patients so that they receive ethical and quality care. We recommend evaluating the experiences of professionals from fields not considered in this study. Include Primary Health Care, since it is a different type of care, in which measures have been taken that, could help improve the quality of care. We also recommended that skills regarding inclusion and access to health care for deaf and disabled people should be incorporated during university studies ♦
